# Ions Speciation at the Water–Air Interface

**DOI:** 10.1021/jacs.3c00517

**Published:** 2023-05-04

**Authors:** Takakazu Seki, Chun-Chieh Yu, Kuo-Yang Chiang, Alessandro Greco, Xiaoqing Yu, Fumiki Matsumura, Mischa Bonn, Yuki Nagata

**Affiliations:** † Max Planck Institute for Polymer Research, Ackermannweg 10, Mainz 55128, Germany; ‡ Graduate School of Science and Technology, Hirosaki University, Hirosaki 036-8561, Aomori, Japan

## Abstract

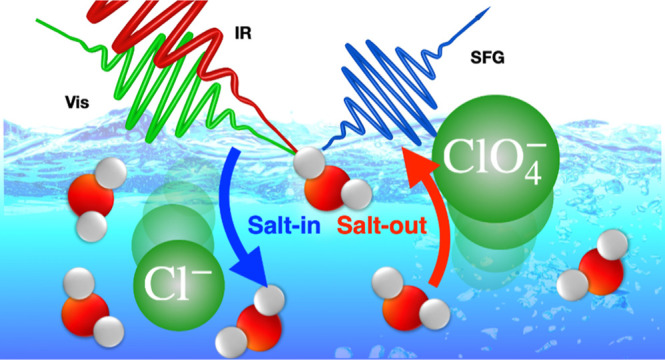

In typical aqueous systems, including naturally occurring sweet and salt water and tap water, multiple ion species are co-solvated. At the water–air interface, these ions are known to affect the chemical reactivity, aerosol formation, climate, and water odor. Yet, the composition of ions at the water interface has remained enigmatic. Here, using surface-specific heterodyne-detected sum-frequency generation spectroscopy, we quantify the relative surface activity of two co-solvated ions in solution. We find that more hydrophobic ions are speciated to the interface due to the hydrophilic ions. Quantitative analysis shows that the interfacial hydrophobic ion population increases with decreasing interfacial hydrophilic ion population at the interface. Simulations show that the solvation energy difference between the ions and the intrinsic surface propensity of ions determine the extent of an ion’s speciation by other ions. This mechanism provides a unified view of the speciation of monatomic and polyatomic ions at electrolyte solution interfaces.

## Introduction

Surface propensities of ions and ionic molecules at the water–air interface play a pivotal role in aerosol growth,^1^ atmospheric chemistry,^2^ and on-water chemistry.^3,4^ At the water–air interface, the density of water is reduced, affecting both the level of ion hydration and the screening of charges through the locally reduced dielectric function^5^ compared with the bulk. Both factors affect the surface propensity of ions. Molecular dynamics (MD) simulations,^6−12^ as well as surface-specific measurements,^13−20^ have revealed that the surface propensity of ions is linked with the Hofmeister series: ions that salt out proteins have stronger hydration and a correspondingly lower surface propensity.

The studies mentioned above have focused on solutions containing a single salt species. On the other hand, a vast majority of ion solutions in the world contain multiple species of salt and ions. For example, seawater contains a variety of inorganic ions such as Na^+^, Cl^–^, and Mg^2+^, together with a very small amount of organic material, including ions. This tiny amount of organic material is believed to be salted out to the sea surface, affecting chemical and physical processes such as sea-spray aerosol^21^ and algal bloom.^22^ However, the distribution of such species at the water–air interface is poorly understood.^23^ The following is a typical question: Can the surface propensity of organic ions be affected by other co-solvated ions? Answering this question allows us to resolve fundamental questions about water on earth; i.e., what is the consequence of the complex ion composition of seawater, and why can unexpectedly large iodine quantities be emitted from the sea surface?^24^

While the complexities of competitive ion adsorption have been investigated,^25−30^ the experimental techniques often have difficulties disentangling the bulk contribution from the surface contribution.^27,29^ Surface-specific spectroscopy such as sum-frequency generation (SFG) spectroscopy can probe the interfacial region selectively and thus has been used for investigating the cooperative ion adsorption at the water–polymer,^26^ water–oil,^25^ and water–surfactant^31,32^ interfaces. However, the technique used in these studies is conventional SFG,^25,26,31,32^ which prohibits us from quantifying the ions’ propensity due to the cooperative behavior of multiple ions because the observables in the conventional SFG can interfere and are not simply additive.^33^

Here, we measure the O–D stretch mode of heavy water D_2_O at the electrolyte solution–air interfaces with the surface-specific heterodyne-detected SFG (HD-SFG) technique^33−36^ by mixing two different species of salts in D_2_O. Thanks to the additivity of χ^(2)^ measured with HD-SFG, we can identify the impact of the salt on the interfaces quantitatively. Our data for various ion combinations show that hydrophobic ions, defined here as ions with a relatively low charge density and associated low solvation energy, are readily speciated (salted out) and are enriched at the interface by decreasing the population of interfacial hydrophilic ions with high charge density and solvation energy. As such, for electrolyte mixtures, ion distributions at the interfaces are determined by cooperative effects. Time-resolved SFG (TR-SFG) spectroscopy is used for probing the ion species that HD-SFG cannot probe. These data further support our notion that hydrophilic ions speciate the hydrophobic ones. MD simulations with two model ion species show that the hydrophobic ions are enriched when the solvation energy difference between hydrophobic and hydrophilic ions becomes large. This study highlights that the surface propensities of ions are significantly controlled by the surface propensity and bulk concentration of other co-existing ions.

## Results

### SFG Signatures of Ions at the Water–Air Interface

For the HD-SFG measurements, we focused the infrared (IR) and visible beams collinearly onto a *y*-cut quartz to generate a local oscillator (LO) signal. A SrTiO_3_ (STO) plate was inserted into the beam path to generate the delay for the LO beam relative to the other beams. These beams were re-focused onto the electrolyte aqueous solutions–vapor interface. The angles of incidence were set to 45°. For the SFG measurement in this work, we used ssp polarization combination, where ssp denotes s-polarized SFG, s-polarized visible, and p-polarized IR beams. NaI solutions were freshly prepared just before the SFG measurements to avoid the oxidization of the iodide ion.^17^ The details of the HD-SFG setup are presented in ref (37).

**Figure 1:**
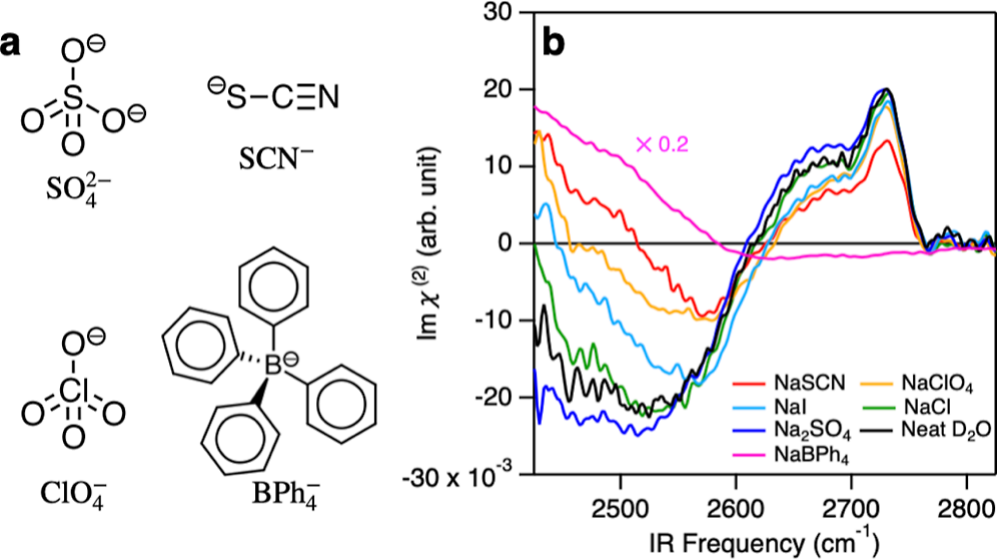
(a) Chemical structures of SO_4_^2–^, SCN^–^, ClO_4_^–^, and BPh_4_^–^ anions. (b) Im χ^(2)^ spectra of the neat D_2_O as well as the D_2_O solution of 3.0 M NaSCN, 1.5 M NaClO_4_, 1.5 M NaI, 3.0 M NaCl, 0.5 M Na_2_SO_4_, and 20 mM NaBPh_4_ at the solution–vapor interfaces. For clarity, the Im χ^(2)^ spectrum for NaBPh_4_ was scaled by a factor of 0.2.

First, we measured the imaginary part of the SFG susceptibility (Im χ^(2)^) of the neat D_2_O as well as the D_2_O solution of 3.0 M NaSCN, 1.5 M NaClO_4_, 1.5 M NaI, 3.0 M NaCl, 0.5 M Na_2_SO_4_, and 20 mM NaBPh_4_ (chemical structures are shown in Figure 1a) at the solution–vapor interfaces. The data are displayed in Figure 1b. The O–D stretch mode region for the neat D_2_O spectrum (Im χ_D_2___O_^(2)^) shows the 2730 cm^–1^ positive peak, 2650 cm^–1^ positive shoulder peak, and 2550 cm^–1^ negative band, consistent with previous reports,^38,39^ arising from the dangling (free) O–D group, the anti-symmetric mode of the D_2_O molecules with two hydrogen-bond donors, and the hydrogen-bonded O–D group, respectively.^40,41^ The addition of salts in the neat D_2_O alters the Im χ^(2)^ spectral features drastically; NaSCN, NaClO_4_, NaI, and NaBPh_4_ make the <∼2500 cm^–1^ Im χ^(2)^ features positive. NaCl (Na_2_SO_4_) elevates (lowers) the <2550 cm^–1^ Im χ^(2)^ negative features. Furthermore, NaSCN, NaI, and NaClO_4_ ions reduce the free O–D feature, and NaBPh_4_ ions completely suppress it. These features are qualitatively consistent with several previous reports,^42,43^ although some details (e.g., for NaCl and NaClO_4_) are different,^43,44^ possibly because of the phase inaccuracy of the previous HD-SFG measurement, as pointed out in ref (45). To our knowledge, no HD-SFG data for NaBPh_4_ have been published previously.

The amplitudes of the Im χ^(2)^ spectra in the hydrogen-bonded O–D stretch region (for example, at 2500 cm^–1^) show the trend that the amplitude is large in the order BPh_4_^–^ > SCN^–^ > ClO_4_^–^ > I^–^ > Cl^–^ > SO_4_^2–^. This inversely follows the Hofmeister series BPh_4_^–^ < SCN^–^ < ClO_4_^–^ < I^–^ < Cl^–^ < SO_4_^2–^,^46,47^ which represents the rank for speciation ability. Because the larger Im χ^(2)^ amplitude in the hydrogen-bonded O–D region indicates the higher surface propensity of ions, the data show that the surface propensity of the ions is linked with the Hofmeister series, as is pointed out theoretically^48^ and experimentally.^43^

### Composition of Ions at Ion Co-solvated Solution Interfaces

**Figure 2:**
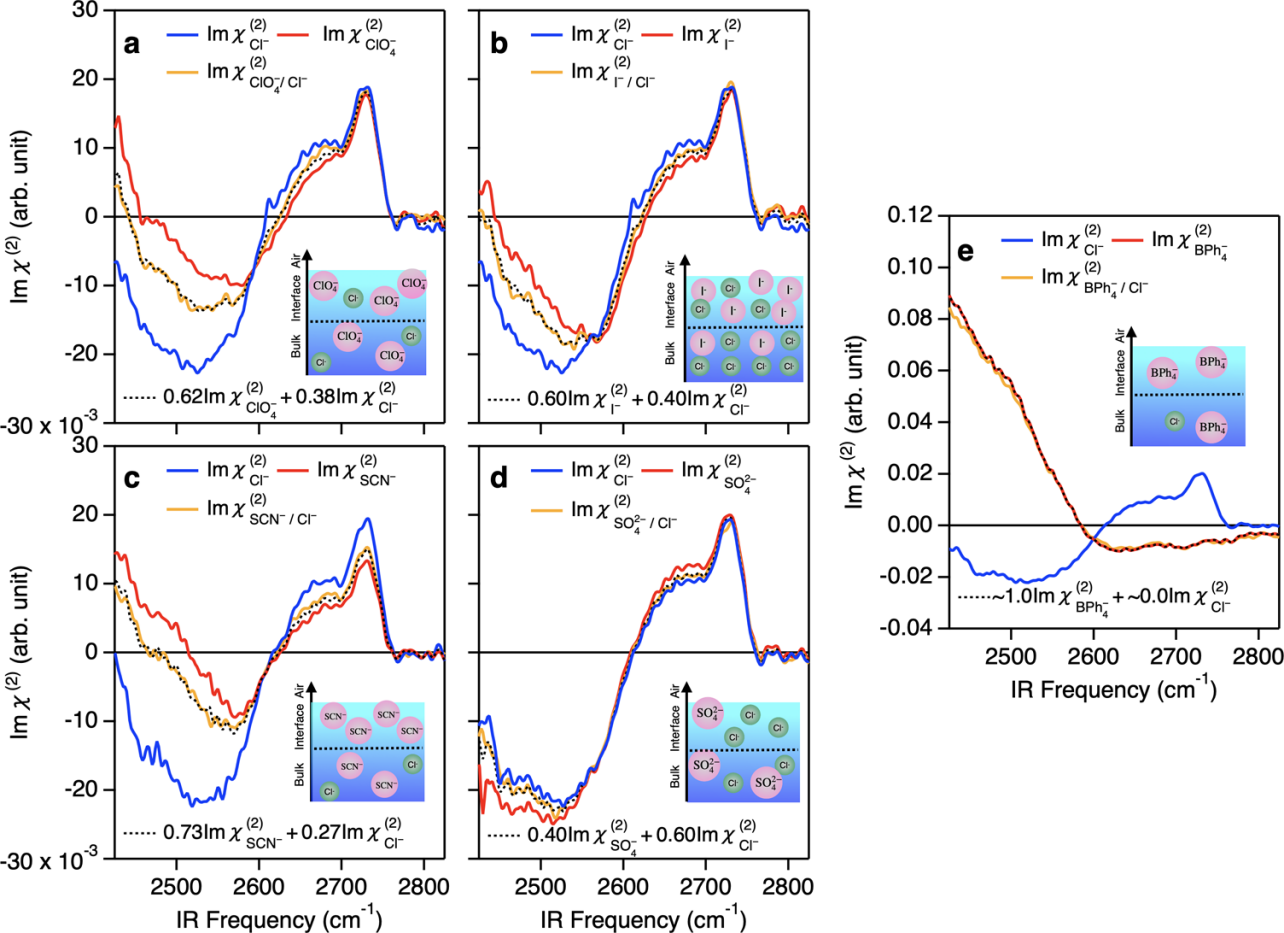
(a–e) Im χ^(2)^ spectra at the D_2_O–vapor interfaces with various salt mixtures: (a) 1.5 M NaCl (), 1.5 M NaClO_4_ (), and 0.75 M NaClO_4_ + 0.75 M NaCl ; (b) 1.5 M NaCl (), 1.5 M NaI (), and 0.75 M NaI + 0.75 M NaCl (); (c) 3.0 M NaCl (), 3.0 M NaSCN (), and 1.5 M NaSCN + 1.5 M NaCl (); (d) 1.0 M NaCl (), 0.5 M Na_2_SO_4_ (), and 0.25 M Na_2_SO_4_ + 0.5 M NaCl (); (e) 20 mM NaCl (), 20 mM NaBPh_4_ (), and 10 mM NaBPh_4_ + 10 mM NaCl (). The black dotted lines represent the fits based on eq 1. The inset schematics depict the putative picture of interfacial ions’ distribution.

Next, we focused on the Im χ^(2)^ spectra for the electrolyte mixtures. We prepared solutions of 0.75 M NaClO_4_/0.75 M NaCl, 0.75 M NaI/0.75 M NaCl, 1.5 M NaSCN/1.5 M NaCl, 0.25 M Na_2_SO_4_/0.5 M NaCl, and 10 mM NaBPh_4_/10 mM NaCl and obtained the Im χ^(2)^ spectra at these solution–vapor interfaces (denoted as , , , , and , respectively). Figure 2a displays the  spectrum, showing a spectral feature similar to the Im χ^(2)^ spectrum of the 1.5 M NaClO_4_ solution () but different from the Im χ^(2)^ spectrum of the 1.5 M NaCl solution (). Apparently, the  spectrum is not the weighted average of the  and  spectra. This indicates that the composition of anions at the interface is not 50% ClO_4_^–^ and 50% Cl^–^; rather, the interfacial region is more heavily populated by ClO_4_^–^ ions. This suggests that the more hydrophobic ClO_4_^–^ ion is speciated at the water–vapor interface by the co-solvated hydrophilic Cl^–^ ion.

To evaluate the interfacial composition of ions, we computed the coefficient  and  by assuming that  can be described by a linear combination of the  and  spectra; i.e., one ion will be replaced by the other ion at the interfaces
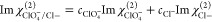
1where we assume that . Note that the validation of this assumption is confirmed in the simulation (see the Supporting Information, Figure S3). The fit result is given in Figure 2a with the coefficients of (, ) = (0.62 ± 0.02, 0.38 ± 0.02), indicating that the D_2_O–vapor interface is dominated by ClO_4_^–^ and the concentration of Cl^–^ decreases.

Subsequently, we performed the same analysis for the other electrolyte combinations. Figure 2b–d displays the , , , and  spectra. The fits using eq 1 provide (, ) = (0.60 ± 0.02, 0.40 ± 0.02), ,  = (0.73 ± 0.02, 0.27 ± 0.02), ,  = (0.40 ± 0.06, 0.60 ± 0.06), and (, ) = (1.00 ± 0.02, 0.00 ± 0.02), where the error bars were estimated from the fits. For the fittings of , , and , we used the Im χ^(2)^ spectra of the 3.0 M, 1.0 M, and 20 mM NaCl solutions for  to have the same concentration of salt, respectively. The relative strength of the coefficients *c* is in the order . This means that not only the surface propensity but also the amount of the speciation of ions in the co-solvated solutions is governed by the Hofmeister series; hydrophobic ions tend to be speciated at the water–air interface due to the presence of the hydrophilic ions. The amount of enhancement of the surface hydrophobic ion concentration is compensated by the amount of decreases in the surface hydrophilic ion concentration; however, it is at a maximum 50% among the anions considered in the Hofmeister series, i.e., except BPh_4_^–^. Note that for ion speciation, nuclear quantum effects are negligible, as is evidenced by the same coefficients obtained in H_2_O solutions (see Supporting Information).

### Direct Probe of the Anion and Cation

**Figure 3:**
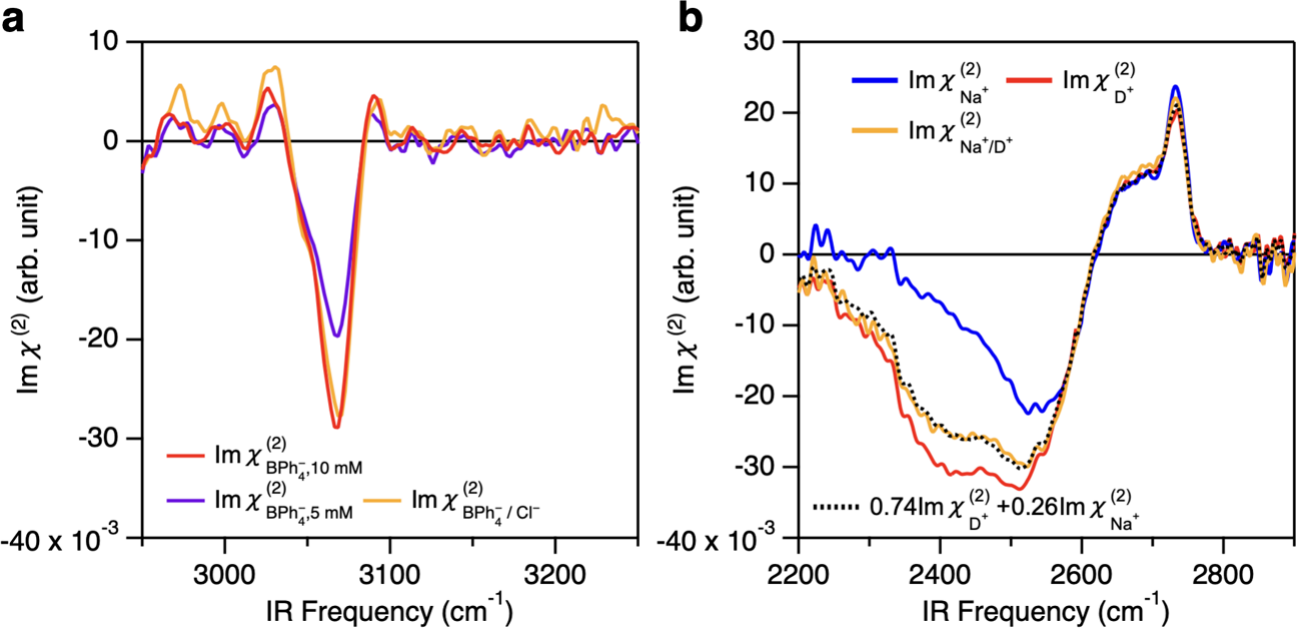
(a,b) HD-SFG measurement of NaBPh_4_ and DCl with NaCl at the D_2_O–vapor interfaces: (a) 5 mM NaBPh_4_ + 5 mM NaCl () and 5 and 10 mM NaBPh_4_ ( and , respectively). (b) 1.0 M NaCl (), 1.0 M DCl (), and 0.5 M DCl + 0.5 M NaCl . The black dotted lines represent the fits based on eq 1.

The above O–D stretch data infer the speciation of hydrophobic anions from changes in the water response, but this does not probe the anion itself directly. To capture the speciation of the anion at the interface, we focused on the BPh_4_^–^ anion and probed the aromatic C–H stretch mode of BPh_4_^–^ at ∼3065 cm^–1^ (Figure 1a);^49,50^Figure 3 displays Im χ^(2)^ spectra of 5 mM NaBPh_4_/5 mM NaCl co-solvated D_2_O solution  together with 5 and 10 mM NaBPh_4_ D_2_O solutions  and  in the aromatic C–H stretch region. These Im χ^(2)^ spectra show a sharp negative peak at ∼3065 cm^–1^ only as there is no resonance for D_2_O. The increase in the concentration of BPh_4_^–^ enhances the amplitude of the C–H peak for the pure NaBPh_4_ solutions. Remarkably, the 5 mM NaBPh_4_/5 mM NaCl co-solvated solution shows the same amplitude of the C–H stretch feature as the 10 mM NaBPh_4_ solution. These data confirm that the hydrophobic ions are enriched substantially due to the chloride ion-induced speciation.

To examine whether this speciation occurs not only for anions but also for cations, we measured the SFG spectra of the mixture of 0.5 M NaCl/0.5 M DCl (), 1.0 M NaCl (), and 1.0 M DCl solutions (). Because D_3_O^+^ is known to be surface active and generates an SFG proton continuum response in the frequency region below the O–D stretch region,^51,52^ one can probe interfacial D_3_O^+^ directly. The data are shown in Figure 3b. The  spectrum and the  spectrum show the D_3_O^+^ feature in the frequency <2300 cm^–1^. The analysis for the linear combination of the spectra gives coefficients of (, ) = (0.74 ± 0.02, 0.26 ± 0.02), clearly demonstrating that in the interfacial region, D_3_O^+^ is disproportionally enriched compared with the bulk due to the presence of Na^+^. This observation manifests that the speciation effect occurs regardless of the sign of the ionic charge.

Above, we showed that the D_3_O^+^ signature is dominant for the  spectrum, while the Na^+^ signature is not clearly present, and thus, it remains unclear whether a large amount of Na^+^ is depleted from the interfacial region. To probe the Na^+^ signature, we carried out the TR-SFG measurement with homodyne detection. Hsieh et al. reported long-lived oscillations (∼1 ns) in time-resolved IR pump/SFG probe signals for different ion solutions,^53^ after the excess vibrational energy is fully relaxed (∼1 ps). This oscillation was assigned to the interference between the SFG signals generated at the water–air interface and that at the shock wave front. The period of the oscillation is the same for different ion solutions since the propagation of the shock wave front is determined by the speed of sounds, which is nearly constant. In contrast, the amplitude and phase of the oscillation differ significantly between different ion solutions. Thus, the long-lived oscillation serves as a reporter of the ion composition at the interface.

**Figure 4:**
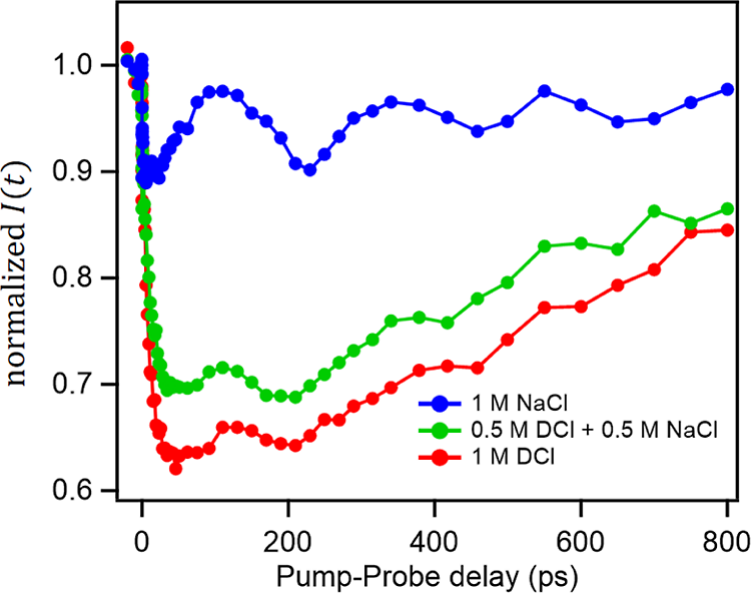
TR-SFG time trace signals of DCl with NaCl at the D_2_O–vapor interfaces. The samples are 1.0 M NaCl, 1.0 M DCl, and 0.5 M DCl + 0.5 M NaCl solutions. The 1.0 M NaCl sample shows strong GHz oscillations, reduced in the presence of excess hydrated protons.

Figure 4 shows the time evolutions of the integrated SFG intensities (*I*(*t*)) of the 0.5 M DCl + 0.5 M NaCl solution, together with the traces of 1.0 M DCl and 1.0 M NaCl solutions, measured at the water–solution interfaces. The samples used in the TR-SFG data are the same as those used for the measurement of Figure 3b. The pure DCl and NaCl solutions exhibit the characteristic dynamics; the NaCl solution shows the characteristic time trace with a large oscillation,^53^ while DCl does not. The mixture solution of 0.5 M DCl + 0.5 M NaCl exhibits a very small oscillation, compared with the pure 1 M NaCl sample. This small oscillation manifests that Na^+^ is excluded from the interfacial region. Note that since the TR-SFG measurement was performed using homodyne detection, so the different signals do not simply add up, prohibiting us from obtaining the coefficients like eq 1. Yet it is apparent from the data that these are consistent with the signal from the DCl/NaCl mixture being dominated by that of the pure DCl response.

## Discussion

**Figure 5:**
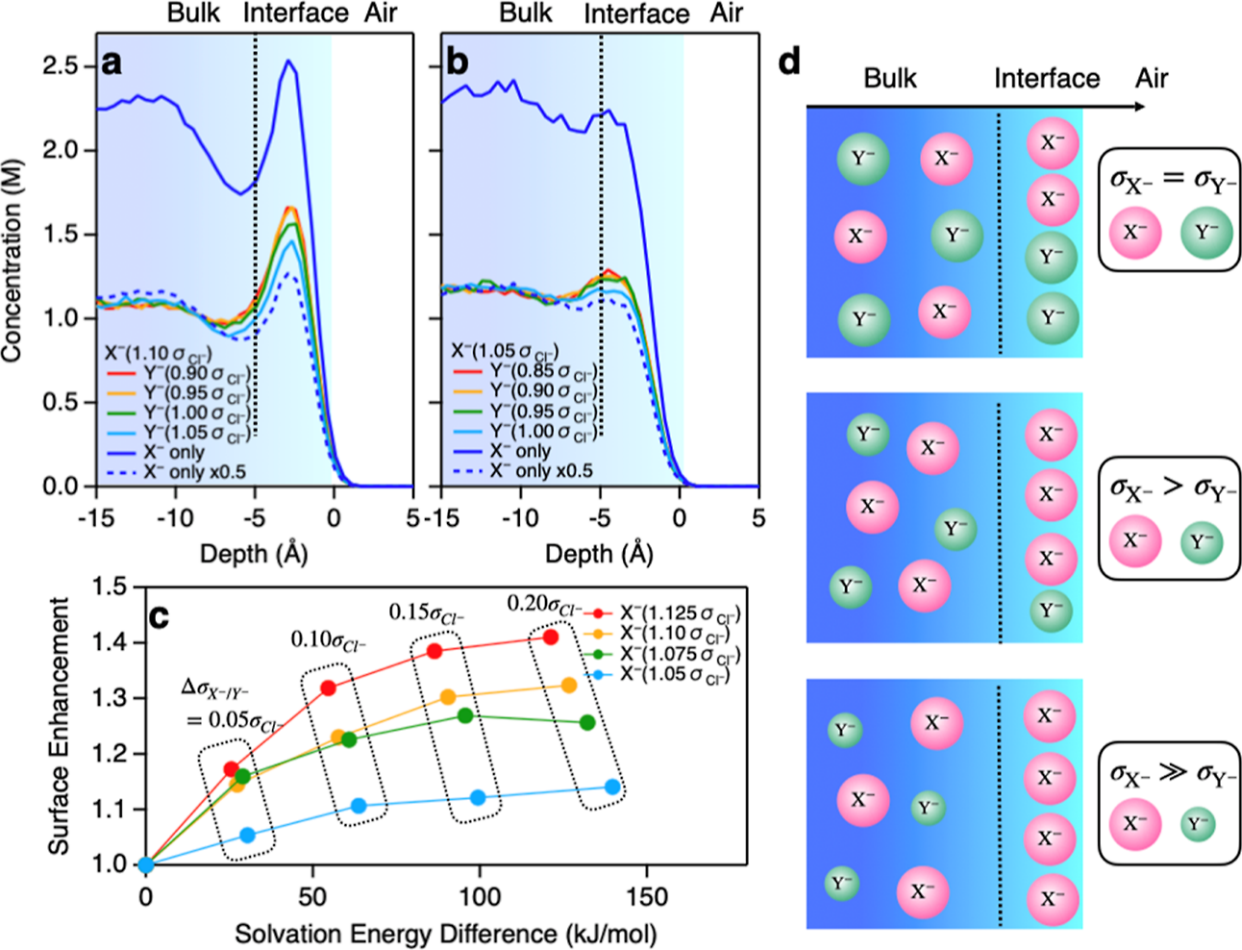
(a,b) Depth profiles of the X^–^ concentration (bulk concentrations: ∼2.4 M for pure NaX and ∼1.2 M each for co-solvated NaX/NaY solutions). *z* = 0 represents the position of the GDS of water. The LJ radii of X^–^ are (a) 1.  and (b) 1.05 . The boundary of the interfacial region and bulk is defined as 5 Å below GDS. (c) Surface enrichment of anion as a function of the solvation energy difference of X^–^ and Y^–^. The surface enrichment is defined as the relative peak concentration in the range −2.4 Å < *z* < 4.4 Å region for X^–^ in mixture solutions with respect to those for pure X^–^ in the blue dotted lines. The peak positions and concentrations were determined based on quadratic fits. . With increasing , the surface enhancement becomes more prominent. Note that the error bars in panels (a–c) are negligibly small (see the Supporting Information). (d) Schematic of the speciation of ions with the variation of LJ radii of ions.

To generalize the idea of the speciation of ions, we simulated the system of the two model salts, NaX and NaY, dissolved in a water solution (∼1.2 M for each anion). X^–^ and Y^–^ have the same Lennard–Jones (LJ) energy minima, while the radii of the LJ potential differ between X^–^ and Y^–^. We set X^–^ to have the larger radius () than the radius of Y^–^ (), making X^–^ more hydrophobic than Y^–^ in the spirits of refs (54) and (55).  and  were set by multiplying a factor to the Cl^–^ radius , while we used the Na^+^ radius for the cation without scaling (see more details in the Supporting Information).^56^ As a reference, we simulated the pure ∼2.4 M NaX solution, where the concentration of NaX equals the sum of the concentrations of NaX and NaY in the co-solvated solutions. We first fixed  and changed . The depth profiles of the X^–^ concentrations are shown in Figure 5a,b for the cases of  and , respectively. The depth axis is denoted as *z*, and the origin point of the *z* axis is the position of the Gibbs dividing surface (GDS) of water.

First, we discuss the case of  (Figure 5a). The comparison between the pure NaX solution and NaX/NaY co-solvated solutions in the bulk region (*z* < −10 Å) shows that the bulk concentration of X^–^ in the co-solvated solution is nearly half of that in the pure solution. In contrast, at the interface (5 Å > *z* > −5 Å), the concentrations of X^–^ in the co-solvated solutions are larger than half of the X^–^ concentration in the pure solution. This confirms that the hydrophobic anion is speciated. The interfacial X^–^ concentration increases with increasing difference between the X^–^ and Y^–^ radii, indicating that X^–^ is more salted out and Y^–^ is more salted in (see also the depth profiles for Y^–^ ion in the Supporting Information). The solvation energy difference of X^–^ and Y^–^ is sizable at 58 kJ/mol when , similar to the solvation energy difference between I^–^ and Cl^–^ of 63 kJ/mol. In this case, the simulation suggests that the peak concentration of X^–^ in the 1.2 M NaX/1.2 M NaY co-solvated solution is 124% of the half of that in the 2.4 M NaX solution at the solution–air interfaces. This inferred surface enrichment is consistent with the interface concentration of (, ) = (0.60 ± 0.02, 0.40 ± 0.02) concluded from the SFG spectra (Figure 2b). This quantitative agreement between the SFG data and simulation manifests that the surface enhancement of the ion occurs because of the difference in the hydrophobicity of the ions.

When looking at the case of  (Figure 5b), one notices that the concentration of X^–^ shows less interfacial accumulation. This is because X^–^ becomes less hydrophobic, the surface propensity of X^–^ is thus rather low, and the surface enrichment is limited. These observations are summarized in Figure 5c,d, where the surface enrichment of X^–^ is plotted as a function of the solvation energy difference of X^–^ and Y^–^ anions. Overall, the surface propensity of X^–^ is enhanced with increasing solvation energy difference between X^–^ and Y^–^, but this is not the only factor in determining the surface enrichment of X^–^. The other key factor is the hydrophobicity of X^–^; if X^–^ is more hydrophobic, X^–^ will be more surface active, as demonstrated by several past pioneering works.^57,58^ This clearly demonstrates that the extent of the speciation of ions is determined not only via the solvation energy difference of X^–^ and Y^–^ but also via the intrinsic surface propensity of X^–^ and Y^–^.

The finding that the intrinsic surface propensity of ions determines the surface enhancement of ions is in line with the observation that such speciation of ions can be seen only when the surface ion population is not saturated. Once the surface ion population is saturated, speciation no longer occurs. This is experimentally confirmed, for example, when the BPh_4_^–^ ion concentration exceeds 10 mM (see Supporting Information), suggesting that the intrinsic surface propensity of ions also matters.

We note that, although the simplified model of ions neglects the impact of the shape of the ions, simulations for SCN^–^, SCN^–^/Cl^–^ mixture, and Cl^–^ solution reveal that ion shape is not a major factor. The data and the discussion are given in the Supporting Information.

Now, we review our results in the context of previous literature. The strong speciation of the charged species has previously been reported for charged surfactants.^31,32,59^ Surfactants are amphiphiles containing a hydrophobic part, which prefers to be exposed to the air at the water–air interface. The strong speciation is the result of the hydrophobicity of surfactant molecules as well as the large solvation energy gap between the dissolved salt and the surfactants. Similarly, strong speciation is observed for SCN^–^ and BPh_4_^–^ anions. These ions are recognized as hydrophobic ions, owing to their relatively low charge density and associated low solvation energy. This hydrophobicity enhances the speciation effect. On the other hand, the speciation is moderate for the pairs of NaI/NaCl, NaClO_4_/NaCl, and Na_2_SO_4_/NaCl. The solvation energy difference between Cl^–^ and I^–^, ClO_4_^–^, and SO_4_^2–^ ions is moderate since these are less hydrophobic than SCN^–^ and BPh_4_^–^.

Our experimental data show that co-solvated ions can salt out other ions, and we generalized our finding based on the simple model ions. Our generalization suggests that this speciation occurs not only for the typical ions but also for the charged molecules. In fact, it has been reported that hydrophobic anions such as bromide and iodide and surfactants/lipids tend to appear at the interfaces upon the addition of salt.^26,27,29,31,32,59,60^ Some of these pioneering works explain this phenomenon, which may be relevant for Jones–Ray effect, by the charge-neutralization/hydrophobization^31,60^ and by the difference in thermodynamic factors such as ion hydration.^26,27,29,30^ In line with refs (26)(27)(29), and (30), our quantitative comparison between HD-SFG data and simulations suggests that this phenomenon can be understood in the framework of the above model: the more hydrophobic anions/surfactants tend to appear at the interface because the hydrophilic ions generated by dissolving salt into water push the hydrophobic anion/surfactant out of the bulk away to the interfaces. The current mechanism can also explain why organic compounds, including volatile organo-iodine compounds, are contained in a sea spray with a high concentration.^61,62^

In summary, we have examined the ion composition at the water–air interface in the presence of pairs of co-solvated ions using HD-SFG and TR-SFG. HD-SFG spectra show that more hydrophobic (hydrophilic) ion tends to be salted out (in). The HD-SFG spectra reveal the tendency of the speciation of ions following the Hofmeister series, and the enhanced surface propensity of the hydrophobic ions are compensated by the hydrophilic ions. The amount of the enhanced surface propensity of the hydrophobic anions due to the presence of the hydrophilic anions is at most 50% among the anions considered in the Hofmeister series. The model simulation indicated that the extent of the speciation is governed by two factors: the solvation energy gap between the ions and the hydrophobicity of the ions. When the solvation energy gap is larger, the speciation is more apparent. The speciation is further enhanced for more hydrophobic ions. This mechanism, corroborated by the quantitative agreement between HD-SFG data and simulations, provides a unified, quantitative view of the speciation of ions and the enhanced surface propensity of the surfactant/lipid in the presence of ions.

## Materials and Methods

### Sample Preparation

Sodium iodide (>99.5%) was purchased from Alfa Aesar. Sodium thiocyanate (99.99%), sodium tetraphenyl borate (99.5%), and D_2_O (>99.9%) were obtained from Sigma-Aldrich for the HD-SFG measurements. For the TR-SFG experiments, D_2_O (99.9%) was obtained by Eurisotop, and HCl (37%) was obtained from VWR. Sodium perchlorate, anhydrous (>98%), was obtained from Thermo Scientific. Sodium chloride (≥99.5%) and sodium sulfate (≥99%) were purchased from Carl Roth GmbH. Sodium chloride was baked at 500 °C for 8 h before use. Other materials were used as received. The DCl solution was prepared by mixing the HCl solution into D_2_O. To avoid the oxidation of iodide ions and BPh_4_^–^ ions, we dissolved the sodium iodide salt into D_2_O under N_2_ atmosphere and in a dark room just before SFG experiments.

### HD-SFG Measurement

We used a collinear beam geometry using a Ti/sapphire regenerative amplifier (Spitfire Ace, Spectra-Physics, centered at 800 nm, ∼40 fs pulse duration, 5 mJ pulse energy, 1 kHz repetition rate). A part of the output was used to generate a broadband IR pulse in an optical parametric amplifier (Light Conversion TOPAS-C) with a silver gallium disulfide (AgGaS_2_) crystal. The other part of the output was directed through a pulse shaper consisting of a grating-cylindrical mirror system to generate a narrowband visible pulse with a bandwidth of ∼10 cm^–1^. The IR and visible beams were first focused on a 20 μm thick *y*-cut quartz plate to generate an LO signal. Then, these beams were collinearly passed through a 5 mm thick STO plate for the phase modulation and were focused on the sample surface at angles of incidence of 45° for visible and IR pulses, respectively. The SFG signal from the sample interfered with the SFG signal from the LO, generating the SFG interferogram. The SFG interferogram was dispersed in a spectrometer and detected by a liquid-nitrogen cooled CCD camera. The complex-valued second-order nonlinear susceptibility (χ^(2)^) from the samples were obtained via the Fourier analysis of the interferogram and normalization by that from a *z*-cut quartz crystal. The measurements were performed with the ssp (denoting s-, s-, and p-polarized SFG, visible, and IR beams, respectively) polarization combination. Using D_2_O rather than H_2_O provides us with improved sensitivity since the measurements are not complicated by water vapor. We expect the conclusions drawn here for D_2_O to also be valid for H_2_O.

### TR-SFG Measurement

The details of the TR-SFG setup are noted elsewhere.^63^ In short, we used the homodyne detection with a Ti/sapphire regenerative amplifier (Spitfire Ace, Spectra-Physics, centered at 800 nm, ∼40 fs pulse duration, 10 mJ pulse energy, 1 kHz repetition rate). The SFG signal was guided into a spectrometer and was detected using an Andor Newton EMCCD camera. The IR pump beam was centered at 2500 cm^–1^. To record pump–probe spectra, a chopper blocks every second laser pulse in the pump laser path, and a vibrating mirror separates the pumped and unpumped signal spatially on the CCD camera. We obtained the time evolution of integrated SFG intensities (*I*(*t*)) in the range from 2300 to 2400 cm^–1^. The details of the analysis can be found in the Supporting Information.

### MD Simulation for the Aqueous Solution

We performed the force field MD simulation for the aqueous solution systems with two anion species and a single cation species. The two anion species have different Lennard–Jones (LJ) radii. We used the 25 Å × 25 Å × 150 Å simulation cell, where we contained 1000 water molecules, 40 cations, and 20 anions with a larger LJ radius (X^–^ ion) and 20 anions with a smaller LJ radius (Y^–^ ion). We used the TIP4P/2005 model for the H_2_O molecules^64^ and the force field models of Na^+^ for cations,^56^ while for X^–^ and Y^–^, we used the LJ radii (), which were the LJ radius for the force field model of Cl^–^ () multiplied by the scale factors. For the SCN^–^ force field, we obtained the parameters from ref (65). To constrain the geometry of the water model, we used LINCS.^66^ For describing ion–ion and ion–water interactions, we used the mixing rule based on the Lorentz–Berthelot rules. We used a cut-off distance of 11 Å for van der Waals interactions and the 3D Ewald summation, smooth particle mesh Ewald,^67^ without a specific dipole correction scheme for taking into account the long-range electrostatics. Note that although the simulation and experiment used H_2_O and D_2_O, respectively, the nuclear quantum effects on the speciation of ions are negligible (see Supporting Information).

We prepared the system which has the same LJ radius of the initial configuration generated by using the PACKMOL software.^68^ MD simulations were performed by using the GROMACS software.^69^ We used a 2 fs time step for integrating equations of motion. We ran the total 200 ns MD simulation in the NVT ensemble and used the first 20 ns for the equilibration. The target temperature was 300 K, controlled via canonical sampling through the velocity rescaling method.^70^ The time constant for the thermostat was 1 ps. The trajectories were sampled every 20 ps.

## Supporting Information Available

Sample preparation, details of HD- and TR-SFG measurements, refractive index of the electrolyte solution, analysis of the TR-SFG data, nuclear quantum effects on the ions’ speciation, effect of the ions’ interfacial concentrations on the ions’ speciation, details of the calculation of solvation energy, depth profiles of ions at the water–vapor interfaces, and surface tension measurement (PDF)

## Notes

Correspondence and requests for materials should be addressed to M.B. and Y.N.

## Notes

All data needed to evaluate the conclusions in the paper are present in the paper and the Supporting Information. Additional data related to this paper may be requested from the authors.
