# Supplementary material for: Ions Speciation at the Water–Air Interface
Source: J Am Chem Soc. 2023 May 4;145(19):10622–30. doi: 10.1021/jacs.3c00517 (PMC10197129; doi:10.1021/jacs.3c00517)
Supplement: Supplementary file 1 — ja3c00517_si_001.pdf [file ja3c00517_si_001.pdf]

Supporting Information on:

Ions Speciation at the Water-Air Interface

Takakazu Seki,[1,2,#] Chun-Chieh Yu,[1,#] Kuo-Yang Chiang,[1] Alessandro Greco,[1] Xiaoqing Yu,[1] Fumiki Matsumura,[1] Mischa Bonn,[1,*] and Yuki Nagata[1,*]

1. Max Planck Institute for Polymer Research, Ackermannweg 10, 55128 Mainz, Germany
2. Graduate School of Science and Technology, Hirosaki University, Hirosaki 036-8561, Aomori, Japan

*Corresponding author. Email: nagata@mpip-mainz.mpg.de, bonn@mpip-mainz.mpg.de

# These authors contributed equally.

Sample preparation

Sodium iodide (>99.5%) was purchased from Alfa Aesar. Sodium thiocyanate (99.99%), sodium tetraphenyl borate (99.5%), and D_2O (>99.9%) were obtained from Sigma-Aldrich for the HD-SFG measurements. For the TR-SFG experiments, D_2O (99.9%) was obtained by Eurisotop, and HCl (37%) was obtained from VWR. Sodium perchlorate, anhydrous (>98%) was obtained from Thermo Scientific. Sodium chloride (≥99.5%) and sodium sulfate (≥99%) were purchased from Carl Roth GmbH. Sodium chloride was baked at 500 °C for 8 hours before use. Other materials were used as received. DCl solution was prepared by mixing HCl solution into D_2O. To avoid oxidation of iodide ion and BPh_4^- ion as much as possible, we dissolved sodium iodide salt into D_2O under N_2 atmosphere and in a dark room just before SFG experiments. We poured the salt solution into a PTFE dish with its diameter of 6 cm and measured SFG spectra from the samples. For NaSCN, we observed the emergence of C-H stretch mode arising from surface active contaminants during SFG measurement. As NaSCN decomposes easily at a significantly lower temperature than other inorganic salts do, the NaSCN sample's surface was cleaned using a home-made aspirator in a similar manner to Refs. 1,2.

Heterodyne-detected SFG measurement

We used a collinear beam geometry using a Ti:Sapphire regenerative amplifier (Spitfire Ace, Spectra-Physics, centered at 800 nm, ~40 fs pulse duration, 5 mJ pulse energy, 1 kHz repetition rate). A part of the output was used to generate a broadband infrared (IR) pulse in an optical parametric amplifier (Light Conversion TOPAS-C) with a silver gallium disulfide ($AgGaS_2$) crystal. The other part of the output was directed through a pulse shaper consisting of a grating-cylindrical mirror system to generate a narrowband visible pulse with a bandwidth of ~10 cm^{-1}.

The IR and visible beams were firstly focused onto a 20 μm-thick y-cut quartz plate to generate a local oscillator (LO) signal. Then, these beams were collinearly passed through a 5 mm-thick $SrTiO_3$ plate for the phase modulation and were focused onto the sample surface at angles of incidence of 45° with pulse energies of ~6 μJ and ~3 μJ for visible and IR pulses, respectively. The SFG signal from the sample interfered with the SFG signal from the LO, generating the SFG interferogram. The SFG interferogram was dispersed in a spectrometer (Teledyne Princeton Instruments, HRS-300) and detected by a liquid-nitrogen cooled CCD camera (Teledyne Princeton Instruments, PyLoN). During the measurements, the sample height was corrected based on a height displacement sensor (GL-82, Keyence).

The complex-valued second-order nonlinear susceptibility ($\chi^{(2)}$) from the samples were obtained via the Fourier analysis of the interferogram and normalization by that from a z-cut quartz crystal. The measurements were performed with *ssp* (denoting *s*-, *s*-, and *p*-polarized SFG, visible, and IR beams, respectively) polarization combination.

During the measurement of $NaBPh_4$ sample, the trough was rotated with a speed of ~1.0 cm per sec with respect to the laser irradiation spot. The rotating trough was needed to avoid the heat accumulation, which may cause the Marangoni flow and thus alter the molecular conformation and the interfacial density[3].

Homodyne-detected time-resolved SFG measurement

We used a non-collinear beam geometry using a Ti:Sapphire regenerative amplifier (Spitfire Ace, Spectra-Physics, centered at 800 nm, ~40 fs pulse duration, 10 mJ pulse energy, 1 kHz repetition rate). A part of the output was used to generate a broadband IR pulse in an optical parametric amplifier (Light Conversion TOPAS-C) with a $AgGaS_2$ crystal (15 μJ). A fraction

of the 800 nm output was sent into an etalon to generate a narrow-band visible laser pulse (~15 cm^{-1}, s-polarized, 20 μJ, 810 nm). By overlapping these pulses at the incident angles of 40° and 70° for IR and visible beams at the sample's surface, respectively, a static SFG signal was obtained and dispersed in a spectrometer (Princeton Instruments, Acton SP2300) and detected by an EM-CCD camera (Andor Newton). The square of the second-order nonlinear susceptibility ($|\chi^{(2)}|^2$) from the samples were obtained via normalization by that from a *z*-cut quartz crystal. Similarly, the homodyne-detected SFG measurements were performed with *ssp* polarization combination.

To generate the narrowband pump (p-polarized, ~90 cm^{-1}, full width at half-maximum, fwhm), the idler output of a second TOPAS-C at ~2000 nm was sent into a BBO crystal to generate ≈1000 nm IR pulses. These pulses were overlapped with 800 nm pulses within a $LiNbO_3$ crystal to generate the narrowband pump pulses. By tuning the frequency of the doubled idler and adjusting the phase-matching condition at the $LiNbO_3$ crystal, the IR pump beam can be tuned between 2350 and 2750 cm^{-1}. To perform the time-resolve SFG measurement, the IR pump beam was guided to the sample's surface at the incident angle of 55°. Prior to each pump−probe experiment, the pump−probe delay dependency is measured and defines the instrument response function. The instrument response function typically has a temporal width of about 200 fs.

Refractive index of the electrolyte solution for reflectivity correction of LO beam.

A number of the papers account for the measurement and processing of the HD-SFG data (*33,59,60*). Here, we briefly describe how to correct the amplitude of the HD-SFG spectra ($\chi^{(2)}$) obtained from different electrolyte solutions at *ssp* polarization combination.

$$\chi^{(2)} = \frac{\chi^{(2)}_{ssp,\text{measured,sample}}}{i\chi^{(2)}_{ssp,\text{measured,zqz}}} \frac{r_{q,s}}{r_{sample,s}}, \quad (1)$$

where $\chi^{(2)}_{ssp,\text{measured,sample}}$ and $\chi^{(2)}_{ssp,\text{measured,zqz}}$ are the SFG interferograms obtained from the electrolyte solution-vapor and z-cut quartz-vapor interfaces, respectively. $\frac{r_{q,s}}{r_{sample,s}}$ represents the ratio of reflectivity coefficients between *z*-cut quartz ($r_{q,s}$) and electrolyte solution sample ($r_{sample,s}$) for *s*-polarized LO beam at ~640 nm and ~660 nm for H_2O and D_2O solutions, respectively[4]. This correction allows us to compare the HD-SFG spectra for different electrolyte solutions.

The reflectivity for s-polarized beam (r_s) is given by Fresnel equation:

$$r_s = \frac{n_{vap} \cos\theta_{vap} - n_{sub} \cos\theta_{sub}}{n_{vap} \cos\theta_{vap} + n_{sub} \cos\theta_{sub}}, \quad (2)$$

where n_{vap} and n_{sub} denote the refractive index for vapor and for the electrolyte solution or z-cut quartz. θ_{vap} and θ_{sub} indicate the incident and refracted angles of the beam for each phase, respectively. Here, we used $n_{vap} = 1$, $\theta_{vap} = 45°$, and $n_{sub} = 1.54$ for z-cut quartz[7,8]. Similarly to our previous work[9], the refractive indices of electrolyte solutions and electrolyte solution mixture for LO beam are calculated using $n = n_0 + c_{electro.} n_{electro.} + c_{NaCl} n_{NaCl}$, where n_0 is the refractive index of neat D_2O or H_2O, $c_{electro.}$ is the concentration of the electrolyte such as NaSCN and $NaClO_4$ in the unit of M, c_{NaCl} is the concentration of NaCl in M. $n_{electro.}$ and n_{NaCl} are the salt specific correction factors for the electrolyte and NaCl (0.0089)[10]. For NaI, $n_{electro.}$ is 0.0207[10]. For NaSCN, $NaClO_4$, Na_2SO_4, and HCl, we estimated $n_{electro.}$ to be 0.0165, 0.011, 0.0178, and 0.0082 from the linear fit for the low concentration regime[11–14], respectively. We assumed the variation of refractive index is negligible between 640 nm and 660 nm region.

Analysis of the time-resolved SFG data

To record pump–probe spectra, a chopper blocks every second laser pulse of the pump and a vibrating mirror separates the pumped and unpumped response, sending them onto different sections of the CCD camera. By dividing the pumped and unpumped intensity spectrum (I_{pumped} and $I_{unpumped}$, respectively), one obtains the bleach ($I(t)$)[15]:

$$I(t) = I_{pumped}(t)/I_{unpumped}(t), \quad (3)$$

where t denotes the delay time between the pump and probe IR pulses. I_{pumped} and $I_{unpumped}$ represent the integrated SFG intensity, $I_{pumped}(t) = \int_{\omega_1}^{\omega_2} \left|\chi^{(2)}_{pumped}(\omega, t)\right|^2 d\omega$ and $I_{unpumped}(t) = \int_{\omega_1}^{\omega_2} \left|\chi^{(2)}_{unpumped}(\omega, t)\right|^2 d\omega$, where we set $\omega_1 = 2300\text{cm}^{-1}$ and $\omega_2 = 2400\text{cm}^{-1}$, respectively. Dividing the two intensities allows for the correction of correlated laser-induced uncertainties like spatial, spectral, and temporal drift. To follow the bleach in time, we use an automated delay stage to change the relative time difference between pump- and probe laser pulses with a minimum step size of 50 fs. The total time range is −20 to +800 ps where the pump arrives after (before) the probe for negative (positive) times.

Nuclear quantum effects on the ions' speciation

To examine the impact of the nuclear quantum effects on the ions' speciation, we performed the HD-SFG measurements for the H_2O solutions of NaCl, NaI, $NaClO_4$, and their mixtures, and compared the data with those for the D_2O solutions. The data of the H_2O solutions are shown in Fig. S1. Based on equation 1 in the main text, we obtained the coefficients of ($c_{ClO_4^-}$, c_{Cl^-}) and (c_{I^-}, c_{Cl^-}) for the H_2O solutions. The obtained coefficients are ($c_{ClO_4^-}$, c_{Cl^-}) = (0.64 ± 0.01, 0.36 ± 0.01) and (c_{I^-}, c_{Cl^-}) = (0.62 ± 0.01, 0.38 ± 0.01). Within the errors, these

coefficients are the same as ($c_{\mathrm{ClO_4^-}}$, $c_{\mathrm{Cl^-}}$) = (0.62 ± 0.02, 0.38 ± 0.02) and ($c_{\mathrm{I^-}}$, $c_{\mathrm{Cl^-}}$) = (0.60 ± 0.02, 0.40 ± 0.02) obtained for the D_2O solutions and discussed in the main text. This observation indicates that the nuclear quantum effects on the ions' speciation are negligible.

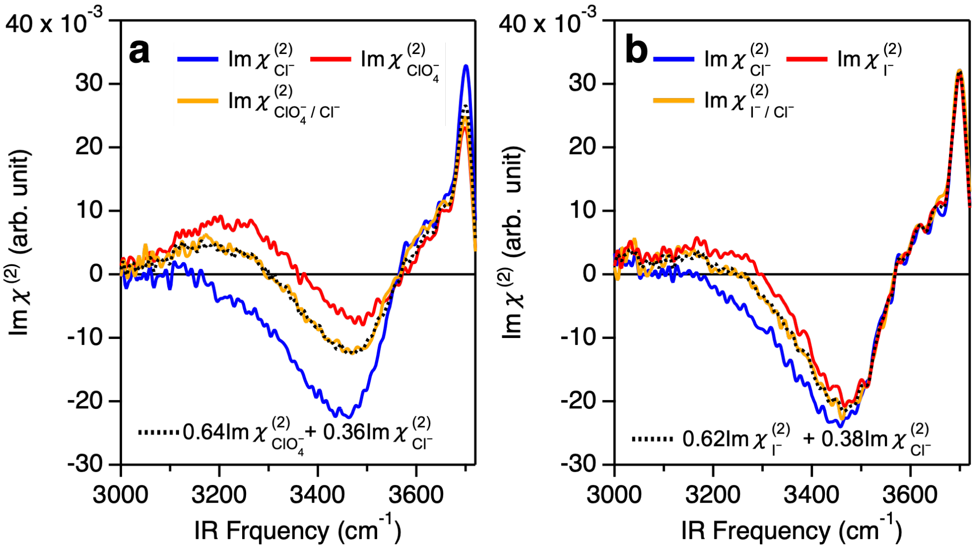

Figure S1: (a) The Im$\chi^{(2)}$ spectra of 1.5 M NaCl ($\chi^{(2)}_{\mathrm{Cl^-}}$), 1.5 M $NaClO_4$ ($\chi^{(2)}_{\mathrm{ClO_4^-}}$), and 0.75 M $NaClO_4$ + 0.75 M NaCl ($\chi^{(2)}_{\mathrm{ClO_4^-/Cl^-}}$) at their H_2O solution-vapor interfaces. (b) The Im$\chi^{(2)}$ spectra of 1.5 M NaCl ($\chi^{(2)}_{\mathrm{Cl^-}}$), 1.5 M NaI ($\chi^{(2)}_{\mathrm{I^-}}$), and 0.75 M NaI + 0.75 M NaCl ($\chi^{(2)}_{\mathrm{I^-/Cl^-}}$) at their H_2O solution-vapor interfaces. The black dotted lines represent the fits based on equation 1 in the main text.

The effect of the ions' interfacial concentrations on the ions' speciation

The surface occupation and the variation of the surface potentials due to the existence of excess ions at the interface may affect the tendency of the speciation of ions. To understand this, by varying the electrolyte's concentrations, we carried out HD-SFG measurements of the $NaBPh_4$/H_2O solution-vapor interface at a higher concentration regime. Note that the data in

the previous section show that H_2O and D_2O will not affect the tendency of the ions' speciation. The sharp negative peak arising from the aromatic C-H stretch mode of BPh_4^- ion appeared at ~3065 cm^{-1} in the $\text{Im}\chi^{(2)}_{BPh_4^-}$ spectrum, which is commonly observed for the concentration range from 10 mM to 100 mM. It is noteworthy that, above 20 mM, ~3065 cm^{-1} peak amplitude in the $\text{Im}\chi^{(2)}_{BPh_4^-}$ spectrum changed negligibly (Fig. S2). As such, for the high concentration region of the interfacial ions, surface occupation and/or the modulation of the surface potential by hydrophobic ions limit the extent of the ions' speciation. In this case, we could not see a significant speciation behavior.

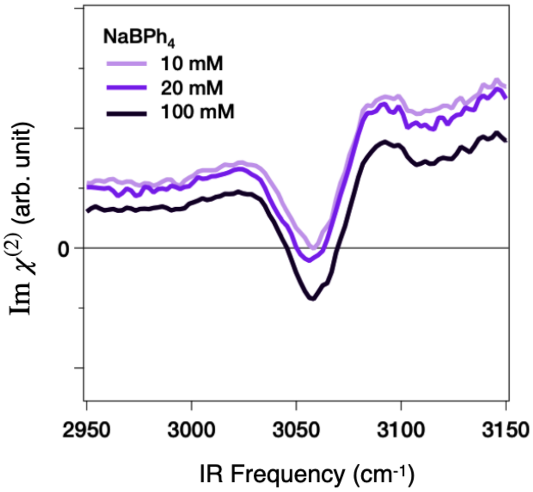

Figure S2: $\text{Im}\chi^{(2)}$ spectra at the $NaBPh_4/H_2O$-vapor interfaces with various concentrations. The amplitude for the ~3065 cm^{-1} peak did not change above 20 mM.

Calculation of solvation energy

We computed the solvation energy by calculating the average potential energy difference of the ion solvated system and pure water system. MD simulations were carried out in the NPT ensemble condition with GROMACS software. The pressure was set to 1 atm, by using the Berendsen barostat with a time constant of 1 ps. The pressure was computed only in the bulk

simulation, where we did not use the long-range correction. The ion solvated system contained 600 water molecules and 1 ion pair. The pure water system consisted of 600 water molecules. We obtained 80 ns MD trajectories, from which we computed the potential energy of the system. The other MD setting is the same as the one used in the MD simulation of the ion solution-air interfaces. The error bars of the solvation energy are typically less than 1%.

Depth profiles of X^- and Y^- ions at the water-vapor interfaces

We computed the distributions of X^- and Y^- ions upon changing σ_{X^-} and σ_{Y^-}. The distribution of X^- and Y^- ions are shown in the solid and broken lines, respectively, in Fig. S3. Figures S3a and S3b show the distributions for $\sigma_{\mathrm{X}^-} = 1.05\sigma_{\mathrm{Cl}^-}$ and $\sigma_{\mathrm{X}^-} = 1.10\sigma_{\mathrm{Cl}^-}$, respectively. First, we focus on the case of $\sigma_{\mathrm{X}^-} = 1.05\sigma_{\mathrm{Cl}^-}$. Upon reducing σ_{Y^-}, the peak concentrations for X^- (solid line) increase and are saturated in the interfacial region, while the concentrations for Y^- (solid line) decrease. The comparison of the data for $(\sigma_{\mathrm{X}^-}, \sigma_{\mathrm{Y}^-}) = (1.05\sigma_{\mathrm{Cl}^-}, 1.05\sigma_{\mathrm{Cl}^-})$ and for $(\sigma_{\mathrm{X}^-}, \sigma_{\mathrm{Y}^-}) = (1.05\sigma_{\mathrm{Cl}^-}, 1.00\sigma_{\mathrm{Cl}^-})$ validates the assumption that the ions are replaced by the other ions when we used the co-solvated ions (see main text). We found the same trend in the case of $\sigma_{\mathrm{X}^-} = 1.10\sigma_{\mathrm{Cl}^-}$ in Fig. S3b. To evaluate the simulation errors, we displayed multiple depth profiles of X^- ion for the case of $\sigma_{\mathrm{X}^-} = 1.13\sigma_{\mathrm{Cl}^-}$ in Fig. S4 by sampling ¼ of whole X-ions for each depth profile. As is clear from the data, the error bars are negligibly small.

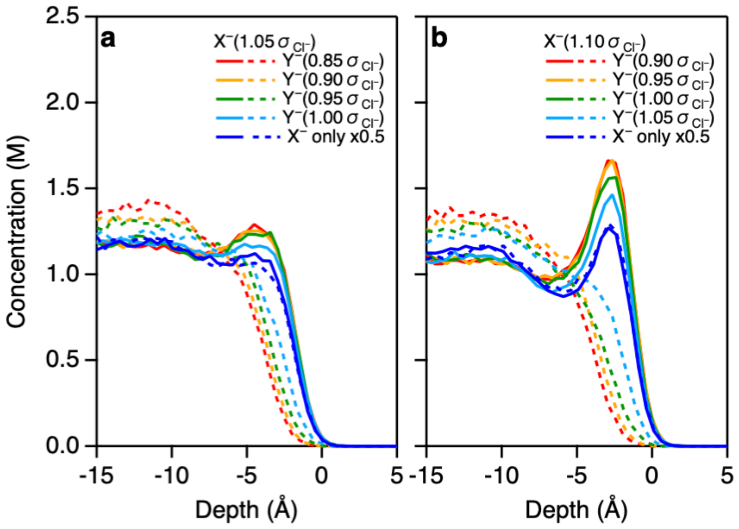

Figure S3: (a, b) Solid and dotted lines represent the density profiles for X^- (a) and Y^- (b) ions, respectively, at the water-vapor interface upon changing relative ion radius σ. $z = 0$ represents the position of the Gibbs dividing surface (GDS) of water.

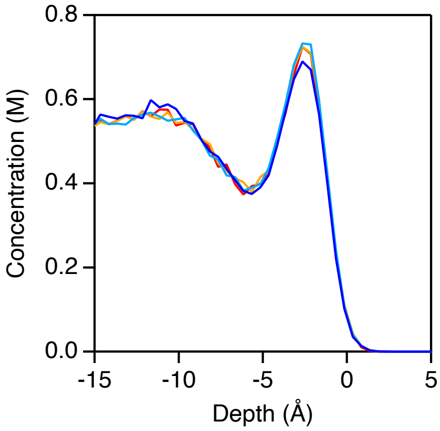

Figure S4: Multiple depth profiles of the X^- concentration at the water-vapor interface for the case of $\sigma_{X^-} = 1.13\sigma_{Cl^-}$. $z = 0$ represents the position of the GDS of water.

Depth profiles of SCN^- and Cl^- ions at the water-vapor interfaces

We computed the distributions of SCN^- and Cl^- ions. The distribution of SCN^- and Cl^- ions in the NaSCN/NaCl mixture are shown in the solid and broken red lines, respectively, in Fig. S5. The data for SCN^- and Cl^- ions in their pure solution are displayed in the blue and orange lines. In line with the discussion in the main text, the distribution of the SCN^- ion was enhanced in the presence of Cl^- ion, suggesting that our discussion based on the simplified solvation model is valid.

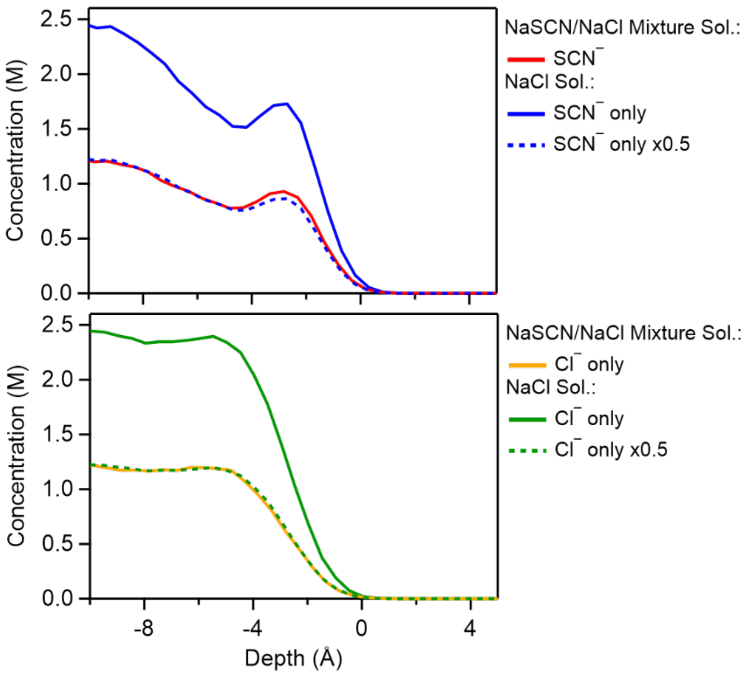

Figure S5: Depth profiles of the SCN^- and Cl^- concentrations (bulk concentrations: ~2.4 M for pure NaSCN and pure NaCl, and ~1.2 M each for co-solvated NaSCN/NaCl solutions). $z = 0$ represents the position of the GDS of water.

Surface tension measurement

To verify that the molecular picture fits the thermodynamic picture, we performed the surface tension measurement of $NaClO_4$, NaCl, and $NaClO_4$/NaCl mixture solutions with a DeltaPi tensiometer (KBN 315 Sensor Head, Kibron Inc.). The data is shown in Fig. S6. The surface tensions of the mixtures showed nonlinear concentration dependence, which is consistent with the concentration dependence of the NaCl aqueous solution in the previous study[16].

When looking closely at the result at the mole fraction of 0.5 which is the condition for our SFG measurement, the surface tension value of the Cl^-/ClO_4^- mixture is almost the average of the pure Cl^- and ClO_4^- solutions. This observation differs from the SFG observation that the spectrum of the mixture is closer to that for the pure ClO_4^- solutions. Such discrepancy might be attributed to the different probed regions of the surface tension measurement and SFG measurement. In fact, SFG probes the interfacial region where the specific molecular orientation of water is present due to the double layer formation[17], while the surface tension measurement probes deeper interfacial region, as the long-ranged electrostatic force substantially influences the surface tension[18]. As such, the SFG spectra are more sensitive to the interfacial structure generated by the ion species.

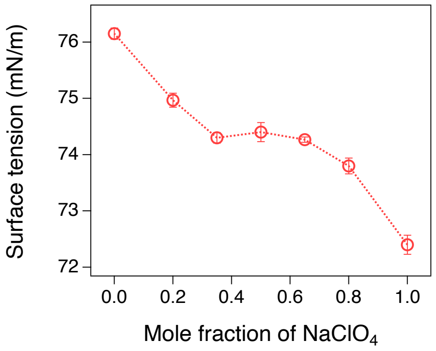

Figure S6: The surface tension data obtained at various mole fractions of $NaClO_4$ in $NaClO_4$/NaCl mixture aqueous solution. The error bars indicate the standard deviation for the multiple measurements. The dotted line is drawn to guide the eye. The total concentration of NaCl and $NaClO_4$ was fixed at 1.5 M.

Supporting References

(1) Nayak, S.; Lovering, K.; Bu, W.; Uysal, A.Anions Enhance Rare Earth Adsorption at Negatively Charged Surfaces. *J. Phys. Chem. Lett.* **2020**, *11*, 4436–4442.

(2) Okur, H. I.; Drexler, C. I.; Tyrode, E.; Cremer, P. S.; Roke, S.The Jones–Ray Effect Is Not Caused by Surface-Active Impurities. *J. Phys. Chem. Lett.* **2018**, *9*, 6739–6743.

(3) Backus, E. H. G.; Bonn, D.; Cantin, S.; Roke, S.; Bonn, M.Laser-Heating-Induced Displacement of Surfactants on the Water Surface. *J. Phys. Chem. B* **2012**, *116*, 2703–2712.

(4) Nihonyanagi, S.; Mondal, J. A.; Yamaguchi, S.; Tahara, T.Structure and Dynamics of Interfacial Water Studied by Heterodyne-Detected Vibrational Sum-Frequency Generation. *Annu. Rev. Phys. Chem.* **2013**, *64*, 579–603.

(5) Shen, Y. R.Phase-Sensitive Sum-Frequency Spectroscopy. *Annu. Rev. Phys. Chem.* **2013**, *64*, 129–150.

(6) Yu, C. C.; Seki, T.; Chiang, K. Y.; Tang, F.; Sun, S.; Bonn, M.; Nagata, Y.Polarization-Dependent Heterodyne-Detected Sum-Frequency Generation Spectroscopy as a Tool to Explore Surface Molecular Orientation and Ångström-Scale Depth Profiling. *J. Phys. Chem. B* **2022**, *126*, 6113–6124.

(7) Ghosh, G.Dispersion-Equation Coefficients for the Refractive Index and Birefringence of Calcite and Quartz Crystals. *Opt. Commun.* **1999**, *163*, 95–102.

(8) Radhakrishnan, T.The Dispersion, Briefringence and Optical Activity of Quartz. *Proc. Indian Acad. Sci. - Sect. A* **1947**, *25*, 260–265.

(9) Piatkowski, L.; Zhang, Z.; Backus, E. H. G.; Bakker, H. J.; Bonn, M.Extreme Surface Propensity of Halide Ions in Water. *Nat. Commun.* **2014**, *5*, 4083.

(10) Max, J. J.; Chapados, C.IR Spectroscopy of Aqueous Alkali Halide Solutions: Pure Salt-Solvated Water Spectra and Hydration Numbers. *J. Chem. Phys.* **2001**, *115*, 2664–2675.

(11) Agrawal, Y. K.; Sabbagh, R.; Sanders, S.; Nobes, D. S.Measuring the Refractive Index, Density, Viscosity, PH, and Surface Tension of Potassium Thiocyanate (KSCN)

Solutions for Refractive Index Matching in Flow Experiments. *J. Chem. Eng. Data* **2018**, *63*, 1275–1285.

(12) Urréjola, S.; Sánchez, A.; Hervello, M. F.Refractive Indices of Sodium, Potassium, and Ammonium Sulfates in Ethanol−Water Solutions. *J. Chem. Eng. Data* **2010**, *55*, 2924–2929.

(13) Lee, K.; Kunjappu, J.; Jockusch, S.; Turro, N. J.; Widerschpan, T.; Zhou, J.; Smith, B. W.; Zimmerman, P.; Conley, W.Amplification of the Index of Refraction of Aqueous Immersion Fluids by Ionic Surfactants. In *Advances in Resist Technology and Processing XXII*; Sturtevant, J. L., Ed.; 2005; Vol. 5753, p 537.

(14) Lewis, J. S.Refractive Index of Aqueous HCl Solutions and the Composition of the Venus Clouds. *Nature* **1971**, *230*, 295–296.

(15) Deiseroth, M.; Bonn, M.; Backus, E. H. G.Electrolytes Change the Interfacial Water Structure but Not the Vibrational Dynamics. *J. Phys. Chem. B* **2019**, *123*, 8610–8616.

(16) Wang, X.; Chen, C.; Binder, K.; Kuhn, U.; Pöschl, U.; Su, H.; Cheng, Y.Molecular Dynamics Simulation of the Surface Tension of Aqueous Sodium Chloride: From Dilute to Highly Supersaturated Solutions and Molten Salt. *Atmos. Chem. Phys.* **2018**, *18*, 17077–17086.

(17) Litman, Y.; Chiang, K.; Seki, T.; Nagata, Y.; Bonn, M.The Surface of Electrolyte Solutions Is Stratified. *arXiv (2022)* 10.48550/arXiv.2210.01527.

(18) Alejandre, J.; Tildesley, D. J.; Chapela, G. A.Molecular Dynamics Simulation of the Orthobaric Densities and Surface Tension of Water. *J. Chem. Phys.* **1995**, *102*, 4574–4583.
